# The structure of *Streptococcus gordonii* surface protein SspB in complex with TEV peptide provides clues to oral streptococcal adherence to salivary agglutinin

**DOI:** 10.1128/iai.00467-25

**Published:** 2026-02-04

**Authors:** Joshua L. Mieher, Norbert Schormann, Sangeetha Purushotham, Veena B. Krishnan, Ren Wu, Manisha Patel, Hui Wu, Champion Deivanayagam

**Affiliations:** 1 Department of Biochemistry and Molecular Genetics, The University of Alabama at Birmingham 164493, Birmingham, Alabama, USA; 2 School of Dentistry, Oregon Health and Science University 212162, Portland, Oregon, USA; University of Illinois Chicago, Chicago, Illinois, USA

**Keywords:** *Streptococcus gordonii*, Antigen I/II, salivary agglutinin, adhesin, *Streptococcus mutans*, GbpC, SspB

## Abstract

*Streptococcus gordonii* is a commensal bacterium in the oral cavity and has many surface adhesins that have been well characterized. SspA/B belongs to the Antigen I/II-like family of proteins, which are well known for their multifunctional adherence capabilities. Most AgI/II-like proteins adhere to salivary agglutinin (also known as glycoprotein 340, Gp340). In an effort to identify the putative binding site on the AgI/II-like family of proteins, we conducted structural studies to determine the V-domain of SspB. In this paper, we report the structure of SspB’s V-domain in complex with a TEV-peptide that was inserted to cleave the histidine tag at the C-terminus after purification. This peptide shared sequence and structural homology with a helical region on the scavenger receptor cysteine-rich (SRCR) domain of Gp340. Our studies with the synthetic peptide PepCD1^SRCR^ show that it inhibits the *Streptococcus mutans* biofilm formation in a dose-dependent manner. A comprehensive comparative analysis of this site with the corresponding sites in the homologous V-domains of *S. mutans* AgI/II and GbpC established that most of these interface residues were conserved. Based on the structural data, mutational analysis was initiated to study the effect of binding-interface residues on the ability of each of these V-domains from *S. mutans* and *S. gordonii* to adhere to salivary agglutinin. Here, we report for the first time the binding site for the V-regions that are distinct among oral streptococci, which provides potential opportunities for therapeutic intervention of pathogenic species.

## INTRODUCTION

Oral streptococci have proteins on their surface that aid in their adherence to the host and tooth surfaces with high affinity and specificity. Among these, the Antigen I/II-like family of proteins is the most well studied. Antigen I/II was originally identified in *Streptococcus mutans* as two different antigens, I and II, and they were thereafter deduced from gene sequencing to be proteolytic products of the same full-length protein (1–4). This protein has been synonymously known as P1, Pac, and SpaP (5–8). Functionally, these AgI/II-like proteins have been characterized to adhere to extracellular matrix (ECM) proteins, such as collagen, laminin, fibronectin, and fibrinogen (9, 10). In addition, their roles in immunology of the oral cavity have also been well documented (1).

Among the adherence propensities, the most well-studied has been AgI/II’s affinity to salivary agglutinin, also known as glycoprotein 340 (Gp340) (11–24). Gp340 is a large glycoprotein that has 13 tandem repeats of the scavenger receptor cysteine-rich (SRCR) domains, and another 14th SRCR sandwiched between two CUB domains, which terminates with a zona pellucida (ZP) domain at its C-terminus (11, 25–28). AgI/II of *S. mutans* was shown to adhere to Gp340 through both the V-domain and the C-domains that are distally separated 50 nm by the elongated fibrillar stalk that is formed by a strong hydrophobic association of the repeats of A- and P-regions, which are synonymously known for their alanine-rich and proline-rich residues (12, 13). In these studies, it was also determined that the V-domain and the C-domains target entirely different sites on Gp340 for their adherence (12, 13). Further studies delineated the SRCR domains on Gp340 as the primary target for adherence, and the high affinity adherence was driven by the metal calcium and glycosylations (29). These studies also determined that the SRCR domains become highly stabilized in the presence of calcium and undergo a conformational change, which are prerequisites for the high nanomolar binding (29). One of the important findings was that the V-domain’s affinity is not affected by the absence of glycosylations, which indicated that these were primarily protein-protein interactions (29).

Many of these investigations were motivated by the necessity to comprehend the function of these adhesins in the pathogenesis of dental caries, a global affliction whose therapeutic alternatives include root canal therapy, cavity restoration, and the prescription of broad-spectrum antibiotics. Many countries are increasingly restricting the repeated use of antibiotics due to the emergence of resistant bacteria. Considering these findings, there is a necessity to devise innovative therapeutic strategies, especially preventive ones, which could aid in resisting the onset or recurrence of dental caries. The adhesive surface proteins of oral streptococci may provide innovative solutions and therefore necessitate a comprehensive understanding of the mechanisms of oral streptococcal adherence to dental surfaces and/or tissues.

In this context, our studies have been focused on deciphering the adhesion modes/mechanisms of oral streptococcal adhesins, and we have structurally and functionally characterized Antigen I/II and the glucan binding protein C (GbpC) (12, 13, 29, 30). An important factor one has to account for in developing inhibitors is to identify if there are specific differences between the pathogenic and commensal species, and this effort led us to investigate SspB (31), which is one of the well-studied AgI/II-like adhesins on the commensal *Streptococcus gordonii*. We present results on the crystal structure of the V-region of SspB in complex with the TEV peptide, which was incorporated to remove the histidine tag at the C-terminus. The serendipitous observation of this peptide bound in a pocket above the calcium binding site led us to identify both through sequence homology and structural modeling a peptide sequence in the SRCR domain. This new peptide PepCD1^SRCR^ exhibited nanomolar affinity to SspB in surface plasmon resonance (SPR) studies. Both AgI/II and GbpC display the V-domain structural motif with inherent variations. *In vitro*, this peptide confers inhibition to biofilm formation in a dose-dependent manner. Given these results, we have analyzed and described the putative binding site in SspB in comparison with AgI/II and GbpC. Our efforts to define this binding site reveals potential opportunities for developing site-specific inhibitors.

## RESULTS

**Fig 1:**
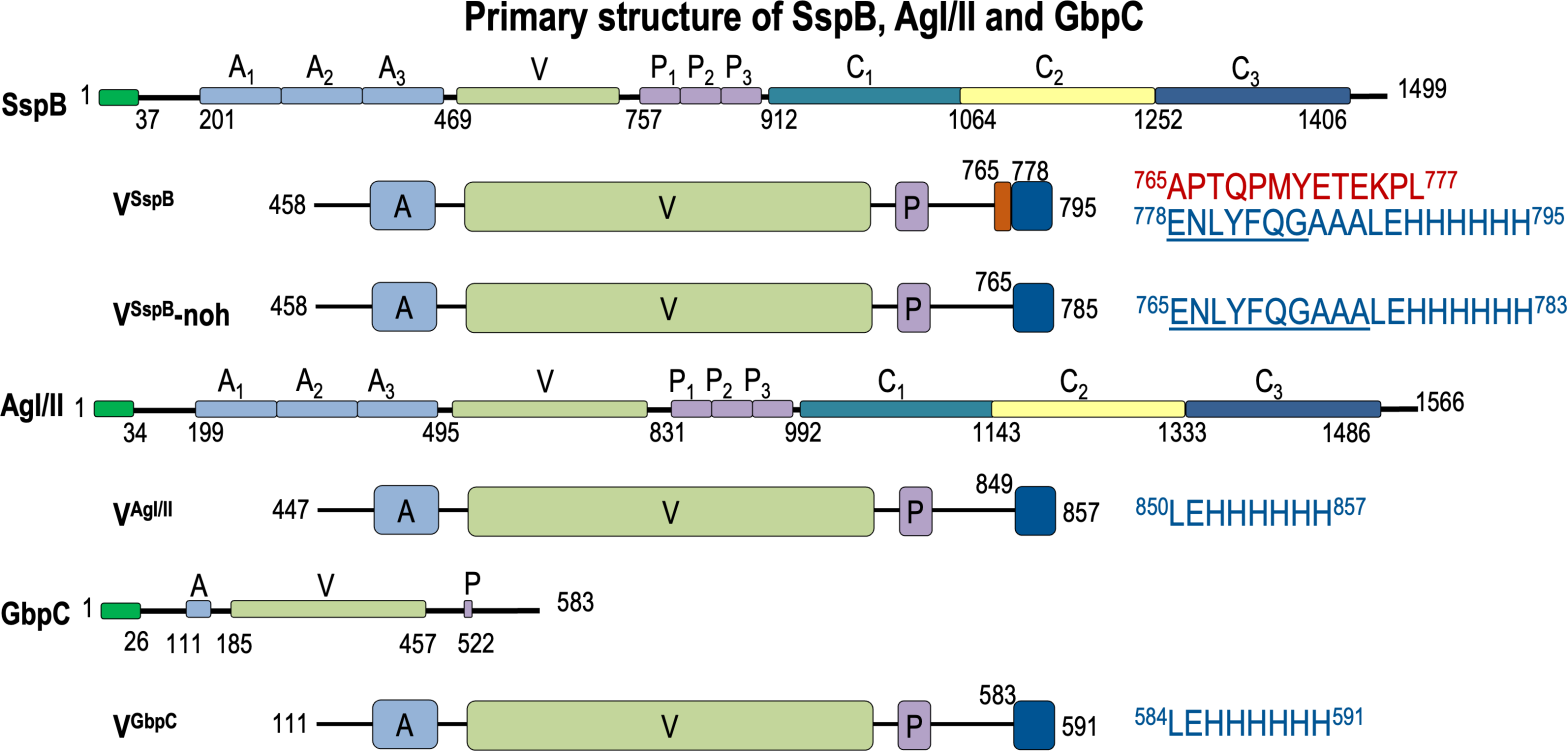
The primary structures of SspB, AgI/II, and GbpC are shown. Each of these proteins has very similar domain arrangements except for GbpC, which only has the V-region. The construction of the various V-regions of SspB, AgI/II, and GbpC is also displayed with the insertion of the TEV cleavage sequence for V^SspB^ and V^SspB^-noh.

For this study on the structural and functional analysis of the variable domains, we cloned, expressed, purified, crystallized, and resolved the structures of V^SspB^ (residues 458–795, 6Q2K), V^SspB^-no-overhang (V^SspB^-noh, residues 458–785, 6Q2L) and the equivalent V^AgI/II^ (residues 447–857, 6TZL) and compared it to the previously reported structure of GbpC (30) (Tables 1 and 2; Fig. 1).

**TABLE 1:** Primers used in this study for cloning V^SspB^ and V^AgI/II^ constructs^*a*^

Construct	Span	Primers: forward/reverse	Restr. enzyme	MW (Da)
V^SspB^	319 (458–776)	ATCCTATCCATGGGGGCCAAATATAAGAAAGAGTTC CTACATGCGGCCGC**GCCCTGAAAATACAGGTTTTC**CAATGGCTTCTCTGTCTCATACAT	*NcoI* *NotI*	37,415
V^SspB^-noh	306 (458–767)	ATCCTATCCATGGGGGCCAAATATAAGAAAGAGTTC CTACATGCGGCCGC**GCCCTGAAAATACAGGTTTTC**TGTTGGTGCAACAGGTTT	*NcoI* *NotI*	35,929
V^AgI/II^	407 (447–849)	TATAAGGATCCAAGCTGATTACGAAGCAAAACTTGCTAAG AATAACTCGAGAGTTGGTTTAGTTGGAGCTGTTGGTTTAACCGG	*BamHI* *XhoI*	44,807

^*a*^ Underlined sequences are the restriction enzyme sites, and in bold are the engineered C-terminal TEV cleavage sites prior to the his-tag.

### Structure of V^SspB^

**Fig 2:**
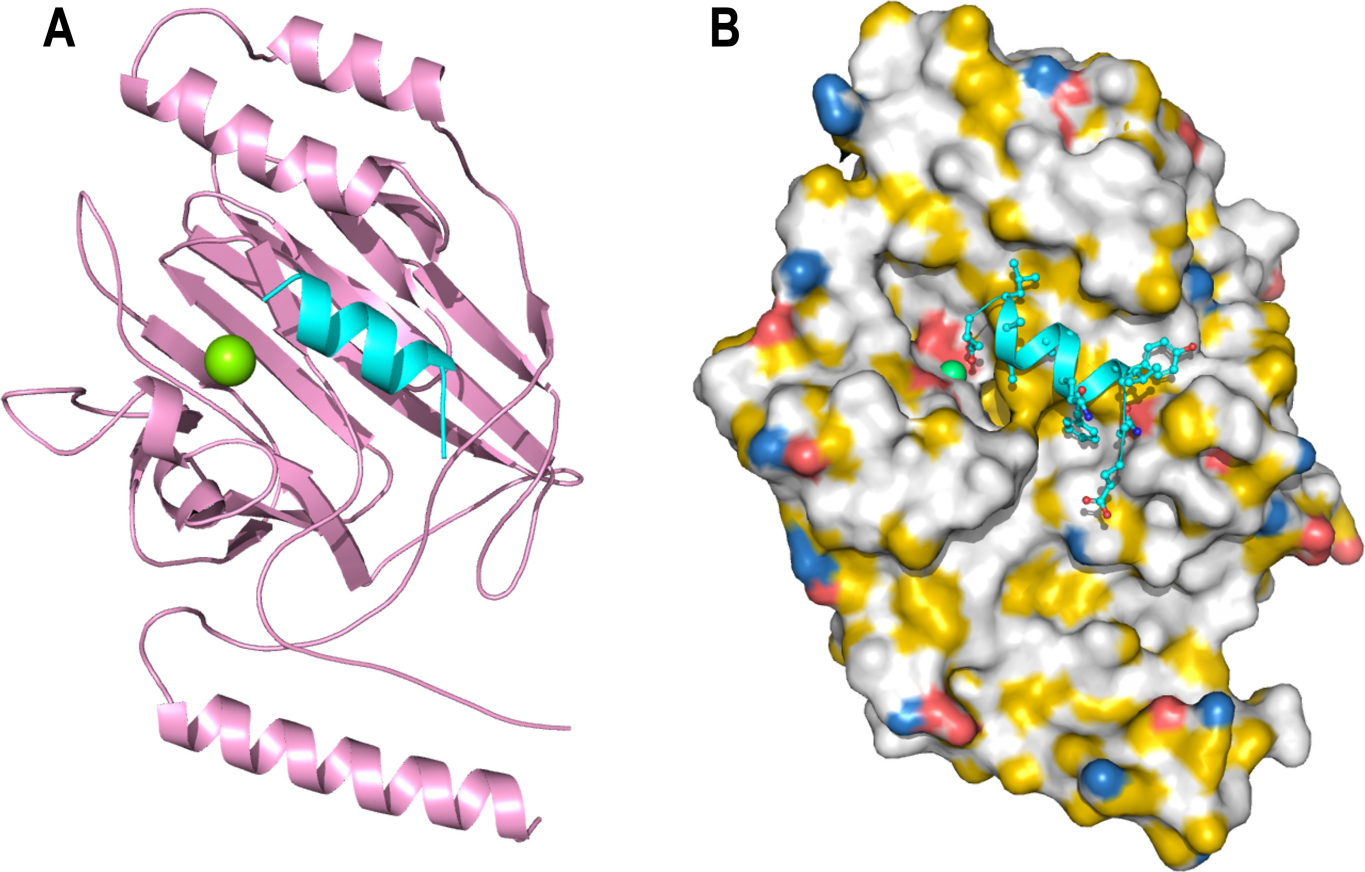
(**A**) The crystal structure of V^SspB^ (pink) resolved at 2.0 Å resolution, showing the TEV^Pep^ in cyan. (**B**) Shown is the V^SspB^ surface using the YRB-coloring scheme, where hydrophobics are shown in white, positive in blue, negative in red, and polar residues in yellow.

The crystal structure of V^SspB^ (residues 458–776) is highly similar to that of the earlier determined V-region structure (32) and superposes with an *rmsd* of 0.37 Å (Fig. 2A). The largest difference between these structures is the presence of the bound peptide in the vicinity of the metal binding site. This was a serendipitous observation resulting from the insertion of a TEV cleavage site at its C-terminal end with an elongated P-region that spanned residues 458–776 (plus 18 residues “ENLYFQGAAALEHHHHHH”) of the SspB protein (UniProt Accession# P16952). After purification, we set up crystallization without removing the histidine tag. Aside from the continuous protein structure (459–764), we discovered an electron density that corresponded to the 12 residue peptide “ENLYFQGAAALE” (778–789), whereas electron density for residues 765–777 was absent. We believe that the 12-residue stretch from the P-region was flexible enough to allow the TEV peptide plus the extra residues from cloning into the pET23d vector to fit into the binding pocket. There are several crystal structures in Protein Data Bank (PDB) that contain the TEV peptide (PDB IDs 4KGI, 6AOK, 3CSI, 5G53, 2H21, 2H23, 2H2E, and 2H2J); we compared them to see if there are similarities, and they all adopt a helical structure.

**Fig 3:**
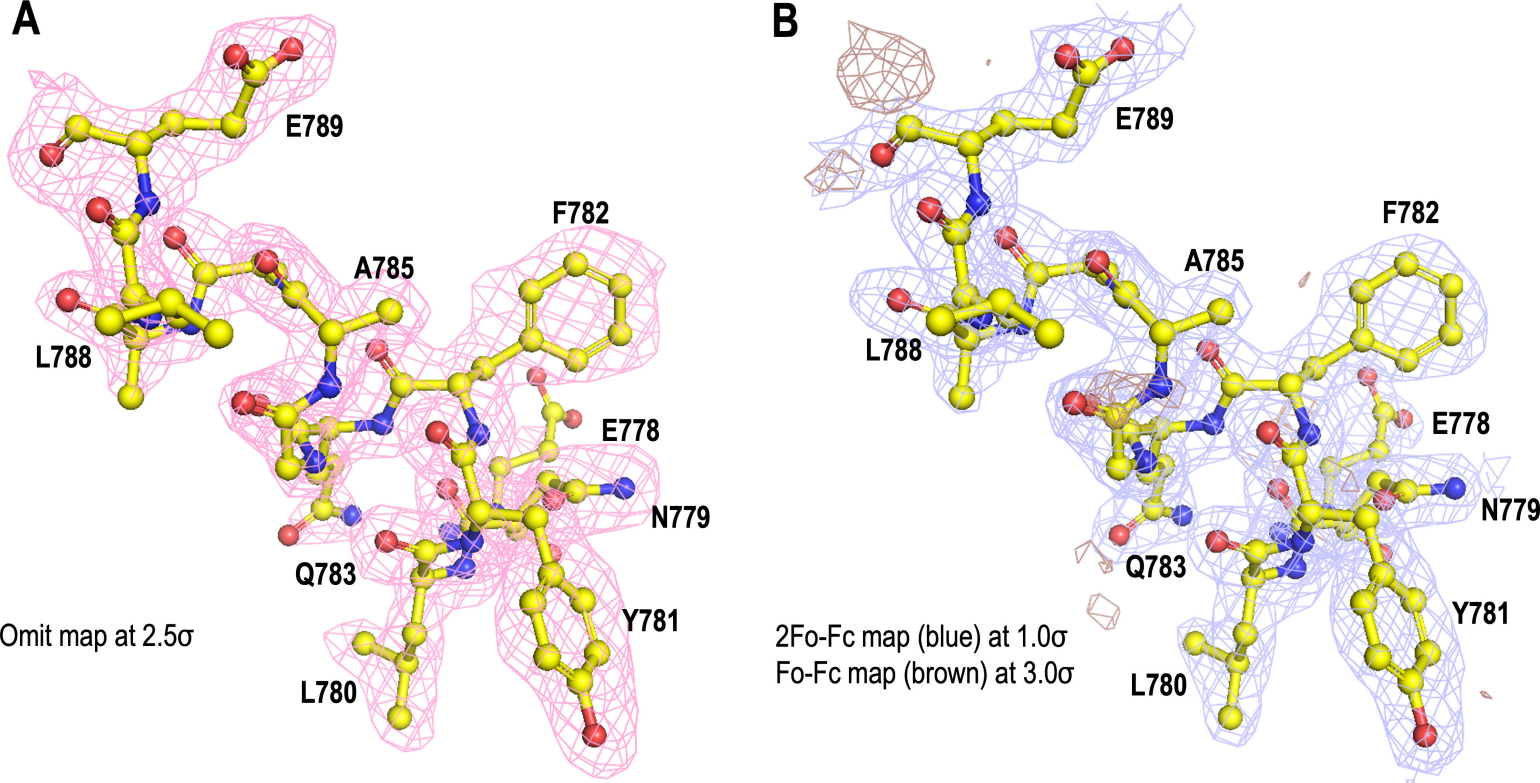
Electron density contours of the TEV^Pep^ (**A**) omit map (pink) contoured at 2.5 σ and (**B**) 2Fo-Fc map (blue) contoured at 1.0 σ and Fo-Fc map (brown) contoured at 3.0 σ.

**Fig 4:**
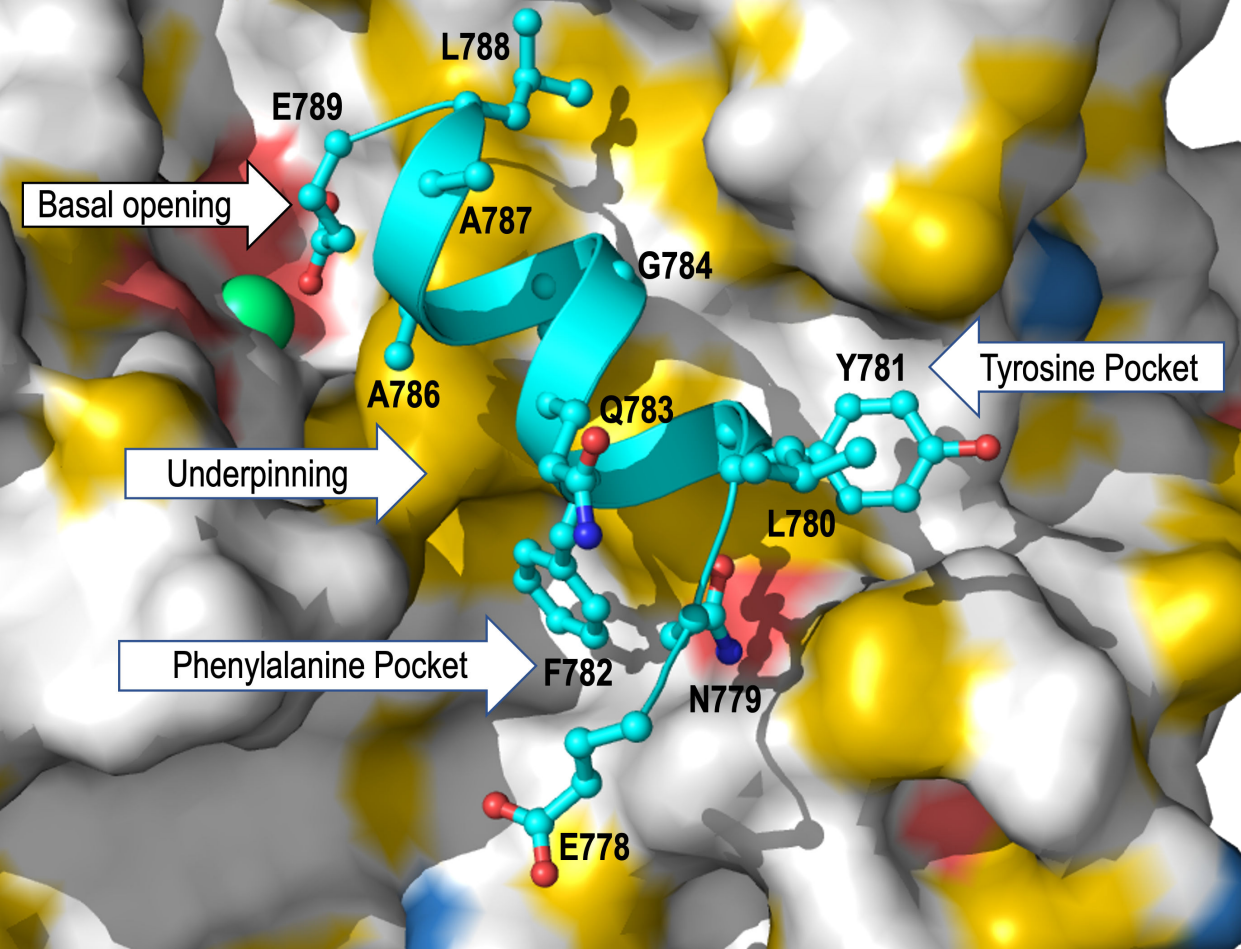
Expanded view of the TEV^Pep : 778^ENLYFQGAAALE^789^ within the V^SspB^ binding pocket. The peptide binding pocket displaying the four distinct regions and the residues that are highly conserved within the structures of V^SspB^, V^AgI/II^, and V^GbpC^ are shown in Table 3. Bolded residues have the most significant reduction in binding to immobilized SRCR_1_ in SPR experiments (Fig. 8).

The omit map and the corresponding 2Fo-Fc, Fo-Fc maps (Fig. 3), and Table 2 describe the refinement parameters of TEV^Pep^ with an average correlation coefficient (CC) of 0.93 and average B-factor of 34.1 Å^2^ compared to the V^SspB^’s CC of 0.90 and average B-factor of 31.4 Å^2^, thus indicating a strongly bound peptide within this pocket. Electrostatic and hydrophobic interactions predominate in this interaction, and the bound peptide has four major regions of contact with V^SspB^, which we classified in Table 3 as (i) basal opening residues: E664, N650, S648, D710, and W709; (ii) underpinning residues: W744 and W613; (iii) phenylalanine pocket residues: A746 and N748; and (iv) tyrosine pocket residues: S606, D607, and V610. The TEV^Pep^ interacting residues within these four regions have been compared with V^AgI/II^ and V^GpbC^ and summarized (Fig. 4; Table 3), with a large number of conserved residues being observed within this binding pocket. The conservation of residues in this binding pocket for the TEV^Pep^ indicates that they might have an evolutionary origin.

**TABLE 2:** CCs and B-factors for the TEV^Pep^

No.	Residue	CC	B-factors (Å^2^)		
			MC	SC	Average
778	GLU	0.74	42.4	51.8	47.6
779	ASN	0.92	30.0	28.5	29.3
780	LEU	0.96	26.6	27.5	27.1
781	TYR	0.97	25.5	24.6	24.9
782	PHE	0.95	26.7	27.0	26.9
783	GLN	0.92	28.7	33.1	31.1
784	GLY	0.96	29.6	0.0	29.6
785	ALA	0.96	29.3	29.1	29.3
786	ALA	0.97	33.3	32.7	33.2
787	ALA	0.96	38.6	37.8	38.4
788	LEU	0.92	44.5	47.0	45.8
789	GLU	0.87	46.6	46.2	46.4
Average B-factor of peptide					34.1
Average B-factor of V^SspB^					30.1

**TABLE 3:** Amino acids in the peptide binding pocket^*a*^

Region	V^SspB^	V^AgI/II^	V^GbpC^
Basal opening	E664	E706	E360
Basal opening	N650	N699	N349
Basal opening	S648	**S697**	S347
Basal opening	D710	**D760**	D408
Basal opening	W709	W759	**W407**
Underpinning	**W744**	W816	W451
Underpinning	W613	**F656**	F304
Phenylalanine pocket	A746	S818	A453
Phenylalanine pocket	N748	**N820**	N455
Tyrosine pocket	S606	T649	H279
Tyrosine pocket	D607	D650	D280
Tyrosine pocket	V610	L653	K283

^*a*^ Bolded residues have the most significant reduction in binding to immobilized SRCR_1_ in SPR experiments (Fig. 8).

**Fig 5:**
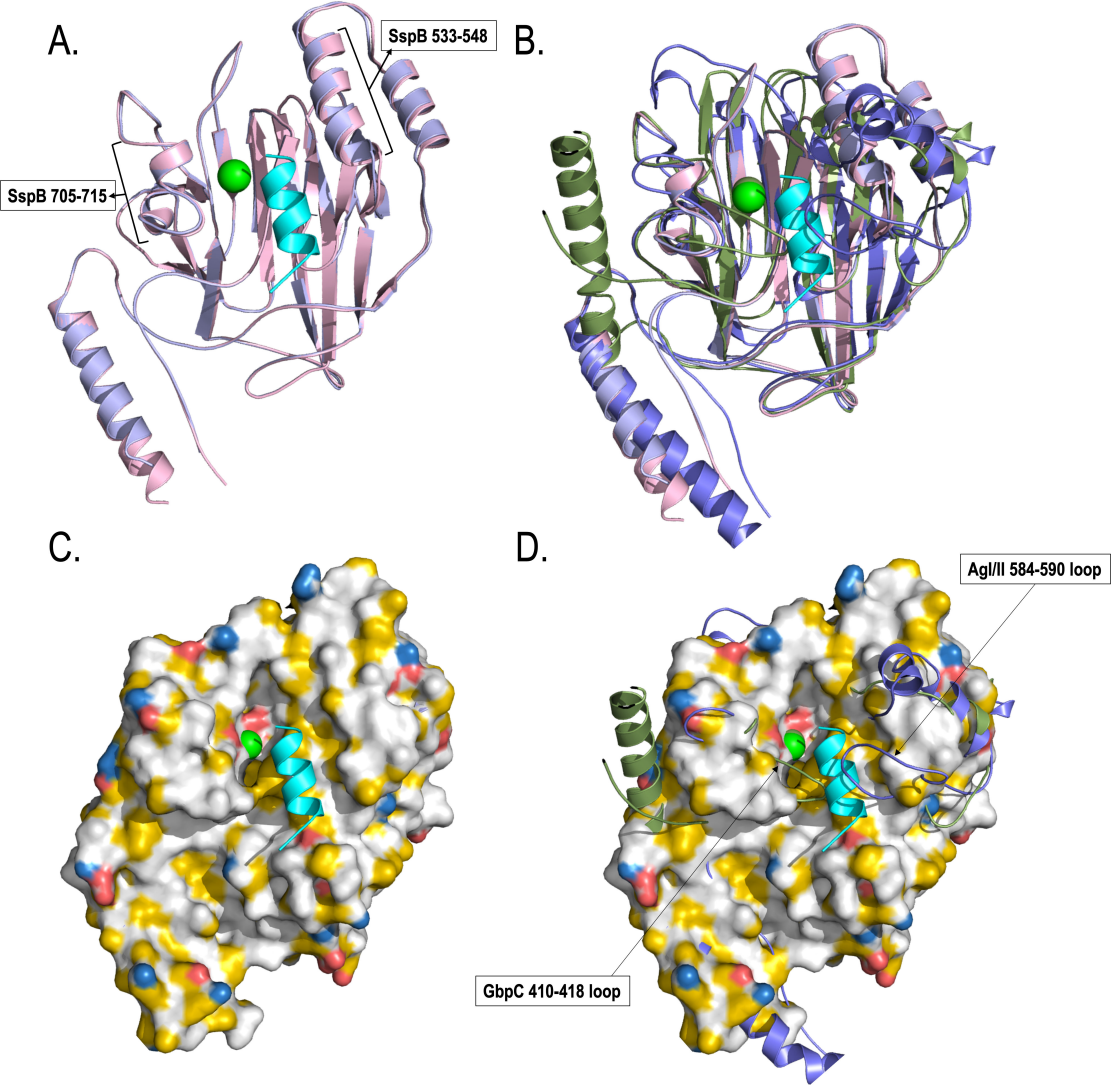
(**A**) Superposition of V^SspB^ (pink) and V^SspB^-noh (slate-blue), where the TEV^Pep^ is shown in cyan, and the calcium ion is shown as a green sphere. The most prominent shift can be observed for the helices on the right side (533–548), where they have shifted up to 1.2 Å to accommodate the binding of the TEV peptide. (**B**) Superposition of V^SspB^ (pink), V^SspB^-noh (slate-blue), V^AgI/II^ (blue), V^GbpC^ (green), where TEV^Pep^ is shown in cyan, and the calcium ion is shown as a green sphere. (**C**) The YRB-hydrophobic surface plot with the bound peptide. (**D**) Significant variations identified in the binding pocket in AgI/II and GbpC, where their loop regions hover over the pocket, underscoring their intrinsic differences.

To identify if there are major changes among these residues within the binding pocket in the absence of the peptide, we designed a new construct V^SspB^-noh (no-overhang). This structure was resolved at 1.8 Å resolution (Fig. 5A), and within the pocket, we observe that residues that interact with the TEV^Pep^ show limited alterations. However, in order to accommodate the binding of the helical peptide, there is a shift on both sides of the binding pocket (Fig. 5A), where both helices (α-helix 533–548 and α-helix 705–710) have shifted by as much as 1.2 Å, and similarly, the loop region (710–715) on the other side of the binding pocket had also expanded by the same amount (Table 4). This movement shows that the binding pocket has the flexibility to expand and contract to accommodate ligand binding.

**TABLE 4:** Residues that display significant deviations between V^SspB^ and V^SspB^-noh^*a*^

Region	N-terminus		Helix 533–548						Helix 705–715		C-terminus
Residue	K463	K464	S509	N531	P532	D533	S548	N550	T712	S713	A762
*rmsd* (Å^2^)	1.35	0.96	1.10	0.92	1.07	0.96	0.99	0.91	1.09	1.24	1.04

^*a*^ Overall *rmsd* between V^SspB^ and V^SspB^-noh is 0.4 Å^2^.

### Comparison with the structures of V^AgI/II^ and V^GbpC^

The structure of V^AgI/II^ superposes with an *rmsd* of 1.10 Å (Fig. 5B). Overall, the structures look very similar, except for specific regions on both sides of the binding pocket, where there are distinct variations. Similarly, V^SspB^ superposes with V^GbpC^ with an *rmsd* of 1.18 Å. Previously, we reported the structure of V^GbpC^, where we observed distinct differences in loop regions that hovered over the dextran binding site (30). Compared to V^SspB^ and its peptide binding region, the loop regions 584–590 in V^AgI/II^ and 410–418 in V^GbpC^ abut inside this binding pocket from opposing sides (Fig. 5B and D). This binding pocket from three different V-domain containing proteins shows major differences indicating that they are tailored to different types of interactions with ligands (Fig. 5D).

### Synthetic peptide adheres to V^SspB^, V^AgI/II^, and V^GbpC^

**Fig 6:**
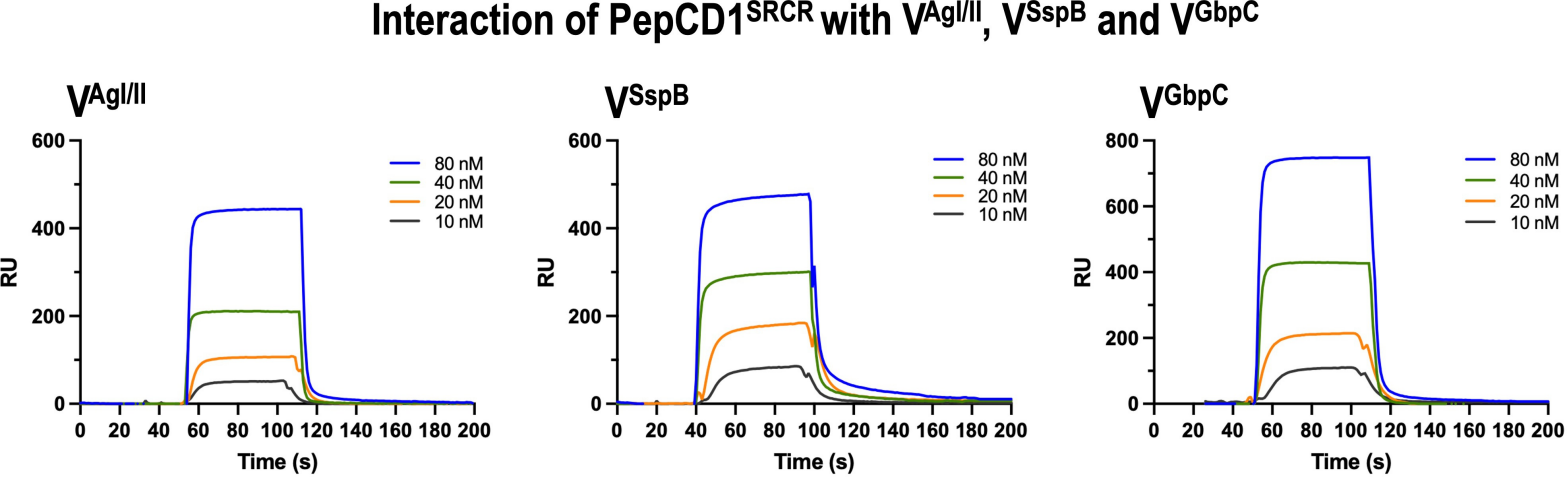
SPR studies on the interaction of the PepCD1^SRCR^ with V^AgI/II^, V^SspB^, and V^GbpC^. The results show that the PepCD1^SRCR^ interacts with nanomolar affinity with the V-regions of AgI/II, SspB, and GbpC.

PepCD1^SRCR^ was designed based on the structural and sequence similarity with the modeled SRCR_1_ domain (Fig. S1) with the sequence “ETNDANVVARQL” as described in “Experimental Procedures.” This peptide interacts with nanomolar affinity as determined by SPR (Fig. 6; Table 5). We chose V^SspB^-noh in these studies since V^SspB^ had expectedly poor binding characteristics (data not shown) due to the presence of TEV^Pep^ at the binding site.

**TABLE 5:** SPR studies on the adherence of PepCD1^SRCR^ peptide

Ligand	Analyte	*K*_*a*_ (1/Ms)	*K*_*d*_ (1/s)	Rmax (RU)	*K*_*A*_ (1/M)	*K*_*D*_ (M)	Chi^2^
V^SspB^-noh	PepCD1^SRCR^	2.29 × 10^6^	0.0314	119	7.29 × 10^7^	1.37 × 10^−8^	8.09
V^AgI/II^	PepCD1^SRCR^	4.10 × 10^6^	0.0864	106	4.74 × 10^7^	2.11 × 10^−8^	6.24
V^GbpC^	PepCD1^SRCR^	7.50 × 10^6^	0.0691	320	1.08 × 10^8^	9.22 × 10^−9^	15.4

### Biofilm assays with the synthetic peptide

**Fig 7:**
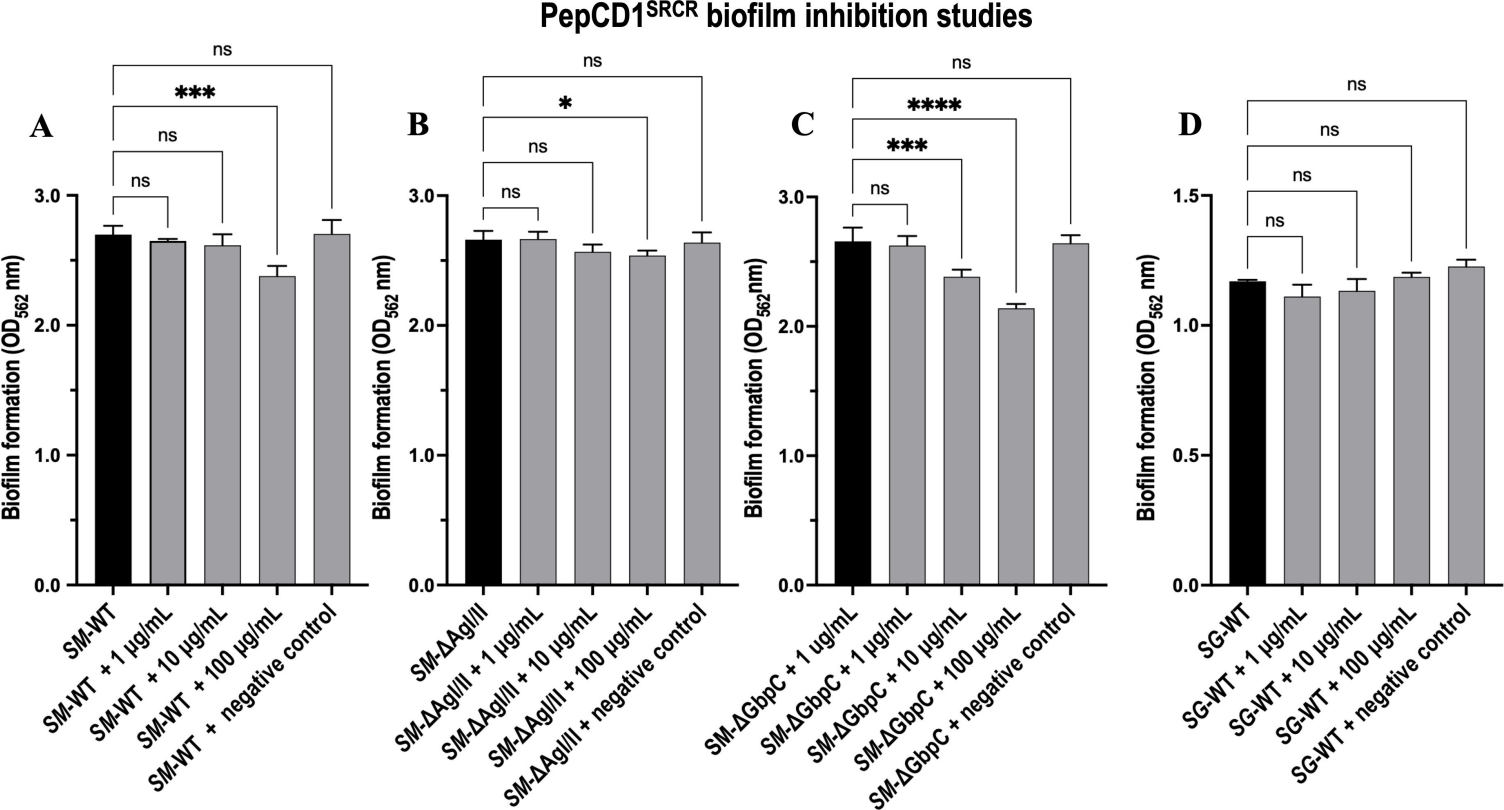
Biofilm inhibition studies with varying concentrations of PepCD1^SRCR^. Graphs show the effect of the peptide (**A**) with wild-type *S. mutans* (*SM*-WT), (**B**) with the AgI/II deletion mutant (*SM*-ΔAgI/II), (**C**) with the GbpC deletion mutant (*SM*-ΔGbpC) and (**D**) with *S. gordonii* (*SG*-WT). The *x*-axis describes the biofilm measured at 562 nm. Statistical analysis was performed by one-way ANOVA and Tukey-Kramer multiple comparisons test using GraphPad Prism, where significance labels are denoted as *P < 0.05, **P < 0.01, ***P < 0.001, ****P <0.0001, ns, not statistically significant.

The supplementation of PepCD1^SRCR^ in biofilm experiments modestly inhibited biofilm formation in a dose-dependent manner (Fig. 7), whereas with *S. gordonii*, there is no significant effect. Both *S. mutans* UA159 AgI/II-deletion mutant (*SM*-ΔAgI/II) and the GbpC-deletion mutant (*SM*-ΔGbpC) exhibit diminished biofilm formation, with a more pronounced reduction observed in the *SM*-ΔGbpC strain. This indicates that the surface proteins on *S. mutans* play a role in biofilm formation and that the pocket to which the peptide binds could be directly involved.

### Site-directed mutagenesis studies

**Fig 8:**
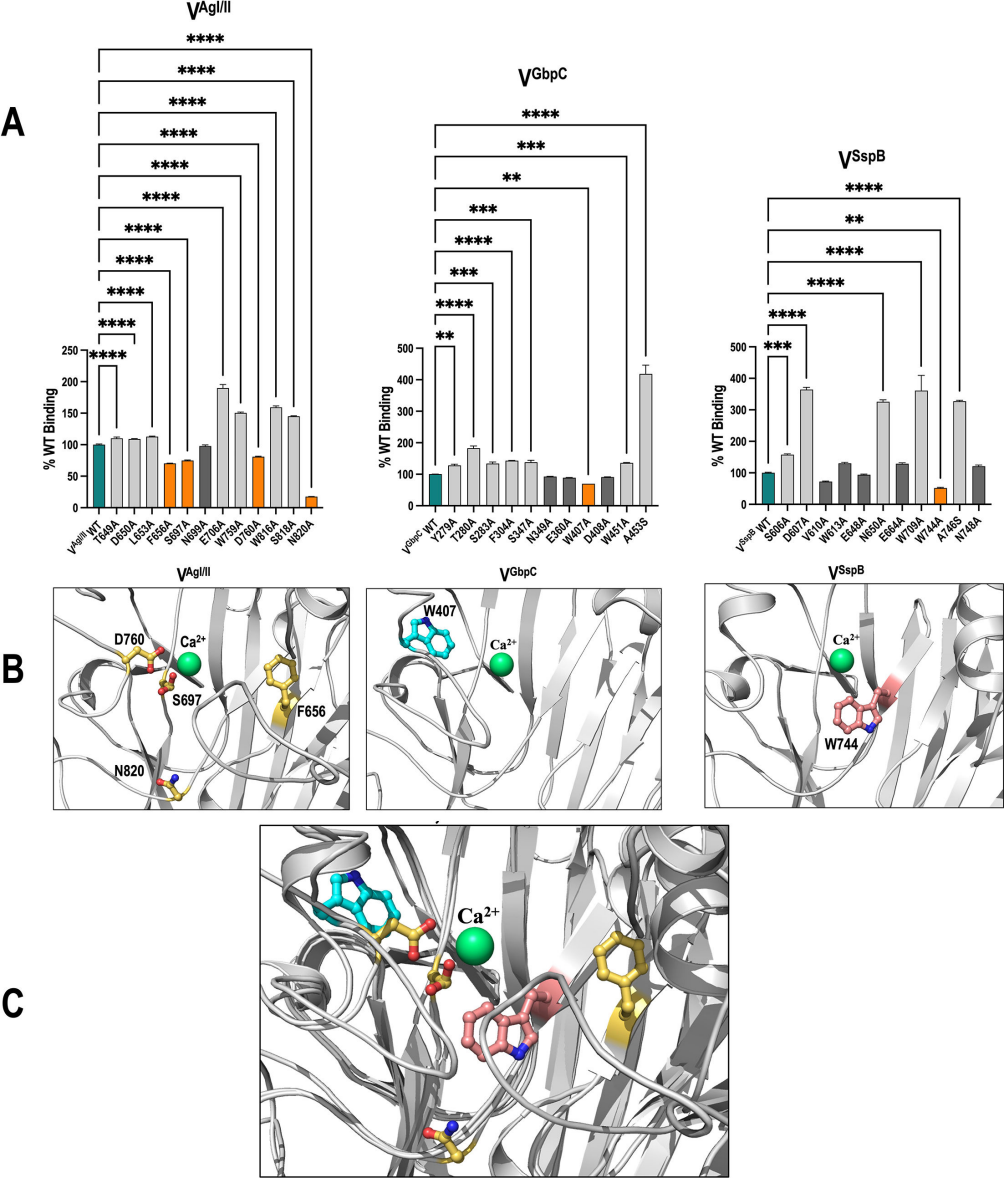
The effect of alanine-substituted mutations on the adherence of V-regions to immobilized SRCR_1_ was analyzed using SPR. (**A**) Mutated residues that resulted in reduced adherence are shown in orange. (**B**) These residues are located in the basal opening (V^AgI/II^ S697A and D760A; V^GbpC^ W407A), underpinning (V^AgI/II^ F656A; V^SspB^ W744A), and the phenylalanine pocket (V^AgI/II^ N820A). (**C**) The superposition of the three proteins illustrates the distinct positions of the residues (V^AgI/II^-yellow, V^GbpC^-cyan, and V^SspB^-pink) that significantly affect their adherence. Statistical analysis was performed by one-way ANOVA and Tukey-Kramer multiple comparisons test using GraphPad Prism, where significance labels are denoted as *P < 0.05, **P < 0.01, ***P < 0.001, ****P < 0.0001.

The 12 residues that came into contact with the peptide or were in close proximity to it, as well as those that exhibited high conservation over this extensive binding area, were selected (Table 3). Each residue was mutated to alanine, and any actual alanines were mutated to serines. The ability of each of these mutants to adhere to immobilized SRCR_1_ was compared to the wild-type (WT) V-domain’s binding (RU) at 8 µM concentration (Fig. S2 to S4). In V^AgI/II^, four mutants S697A, D760A, F656A, and N820A were found to have lower binding than the WT. In V^GbpC^, W407A was the only residue with reduced binding compared to the WT, while in V^SspB^, also W744A was the only residue with reduced binding. Residues whose substitution resulted in reduced binding are bolded in Table 3 and were primarily located in the basal opening and underpinning region of the V-domain’s binding pocket (Fig. 8).

## DISCUSSION

The AgI/II-like proteins on oral streptococci have two regions, a V-region and a C-region, which have been implicated in adherence to host proteins (12, 13, 29, 30). The V-region is present on the distal end of the fibrillar architecture formed by the repeating alanine-rich A-region and the proline-rich P-region, whose alpha-helix and PP-II like helix intertwine to form stable fibrils (12, 13). At the C-terminal end, there are usually three DEv-IgG-like domains that also form an elongated structure, which would be closer to the microbial cell surface (12, 33, 34). While the DEv-IgG domains have now been observed in various bacteria and fungi, the V-domain is uniquely observed in oral streptococci and has been shown to be present in group A streptococci, as in AspA of *Streptococcus pyogenes* (35). Despite numerous studies to date, the binding pocket and the mode of adhesion have yet to be discovered. In this context, we began our studies comparing the differences between pathogenic and commensal streptococci in the oral cavity. Previously, we reported the structure of the V-domain of AgI/II (13) and structural studies on GbpC (30), which is very similar to AgI/II. In GbpC, we identified the dextran binding region and through mutagenesis discovered that the loop regions that hover over this binding pocket in AgI/II (residues 584–590) and GbpC (residues 410–418) had important roles in biofilm formation and aggregation (30). We began with V^SspB^, whose structure was previously reported by Forsgren et al. (32). While their study described a putative binding site and reported that V^SspB^ had no propensity for binding to carbohydrates, we aimed to comprehensively describe the specific amino acids that encompass this binding site. With this goal, we began constructing regions of interest in the V-domain of SspB and, by serendipity, designed a construct with an extended P-region (^765^APTQPMYETEKPL^777^) followed by the TEV cleavage site and the hexa-histidine tag (^778^ENLYFQGAAALEHHHHHH^795^). After crystallizing and resolving the structure, we observed the presence of a helical density within the binding pocket (Fig. 2). Final refinement resolved this density to arise from the TEV^Pep^ (^778^ENLYFQGAAALE^789^) that was introduced for cleaving the 6x histidine-tag. This peptide binding site, although a serendipitous find, provided the basis for all our investigations, specifically in identifying residues that play an important role in adherence to Gp340.

This peptide was found within the same pocket that we had previously identified to be the binding site for glucose/dextran (30) in V^GbpC^. Comparison of the structures of V^SspB^, V^AgI/II^, and V^GbpC^ shows that this binding pocket has many possible modifications in each of these V-domain structures. Among these, V^SspB^ has the flattest surface, whereas the same regions on V^AgI/II^ and V^GbpC^ have very different deeper pockets, indicating that they could have evolved to adhere to various ligands. In the crystal structure of GbpC, we observed higher B-factors compared to surrounding residues for the loop region residues 410–418 and proposed that they might be able to move outward to accommodate the glucans and thereafter move over on top to lock the ligand into place (30). Such a mechanism could also be possible with AgI/II whose 584–590 loop region would provide the locking mechanism (30). Apart from these differences in the loop regions that hover over the binding pocket, we analyzed the peptide interactions and classified them into four different regions (Fig. 4): (i) basal opening, (ii) underpinning, (c) phenylalanine pocket, and (d) tyrosine pocket. Based on the analysis of all V-domains (V^SspB^, V^AgI/II^, and V^GbpC^), 12 residues were chosen for this study (Table 3), and strikingly many of the residues are highly conserved in each of these regions, indicating the existence of an evolutionary origin.

To probe this binding site for its putative role in binding Gp340’s SRCR domains, we initially modeled the SRCR_1_ domain (Fig. S1), and it was eventually confirmed through crystal structures (6SA4 and 6SAN) that a single helical segment exists on all SRCR domains with the consensus sequence “DTNDANVVCRQL.” Based on this finding, we designed the peptide “ETNDANVVARQL” as described in “Experimental Procedures.” Our initial attempts to study the adherence with V^SspB^ resulted in poor binding (data not shown), and we reasoned that the TEV^Pep^ observed in the crystal structure could be occupying the binding site, hence the negative binding result, and therefore designed and purified V^SspB^-noh. We then confirmed that this peptide bound with nanomolar affinity to V^SspB^-noh, V^AgI/II^, and V^GbpC^. Having confirmed that this peptide adheres with high affinity (Fig. 6), we tested the effect of the peptide in biofilm formation studies, where biofilm formation by WT *S. mutans* is modestly inhibited by the peptide in a dose-dependent manner (Fig. 7). The *S. mutans* UA159 AgI/II-deletion mutant (*SM*-ΔAgI/II) exhibits a statistically significant reduction at 100 μg/mL, while the GbpC-deletion mutant (*SM*-ΔGbpC) demonstrates a more pronounced impact. It appears that the peptide effectively affects proteins other than GbpC on the *S. mutans* cell surface, including AgI/II. The same peptide does not confer any effect on *S. gordonii* DL1 strain, thus making the peptide PepCD1^SRCR^ specific toward *S. mutans*. Biofilm studies conducted with *SG-*WT and *SG-*ΔSspB indicate that SspB does not significantly influence biofilm development (Fig. S5). This result is significant, as *S. mutans* carries two V-region containing proteins, which are AgI/II and GbpC, and this peptide seems to block the binding sites and offers modest inhibition. The question would arise why it did not completely abrogate the binding, and the answer lies in the presence of multiple other proteins on the surface of *S. mutans*, which could act as redundant mechanisms that still allow the microbe to adhere to Gp340. This also relates to *S. gordonii*, where 26 LPxTG-anchored proteins have been identified (36), several of which may be Gp340 adherent surface proteins, resulting in the observation of minimal biofilm suppression.

Our recent results for the collagen-binding proteins Cnm (37), WapA, and WapE (unpublished results) show that each one of them adheres to Gp340. This discovery of multiple receptors on *S. mutans* that interact with Gp340 is significant (30), as previous studies on single deletion mutations of AgI/II and/or GbpC did not significantly deter *S. mutans’s* virulence, albeit there were reductions in colonization (38–40). It is here that identification of binding sites on multiple surface proteins present on *S. mutans* and the commensal *S. gordonii* would provide the opportunity to extensively study their modes/mechanisms of binding and comparatively allow the design of inhibitors that would compete specifically with pathogenic strains.

To investigate the role of the residues in the binding sites, a panel of conserved residues in V^AgI/II^, V^GbpC^, and V^SspB^ was selected for site-directed mutagenesis to allow direct comparison of the contributions of homologous residues to the V-domain’s adherence to SRCR_1_ of Gp340. Alanine substitution mutations resulting in decreased binding were found at the basal opening (V^AgI/II^ S697A and D760A; V^GbpC^ W407A) and underpinning (V^AgI/II^ F656A; V^SspB^ W744A) of each of these V-domains (Fig. 8), implying that this common region may be a focal point for inhibitor design. Additionally, V^AgI/II^ of *S. mutans* demonstrated a more pronounced reduction in binding with the N820A mutation in the phenylalanine pocket.

The clear differences in the positions of amino acids that affect adherence to SRCR domains (Fig. 8C) now provide the ability to selectively inhibit V^AgI/II^ residues without affecting *S. gordonii’s* V^SspB^, fostering the potential for the design and development of pathogen-specific inhibitors. Our comprehensive study now offers definitive proof and potential for this method, necessitating the discovery of inhibitors for AgI/II that specifically concentrate on the phenylalanine pocket, where N820 is situated, as it exhibited the greatest disruption in binding. We anticipate that future inhibitor designs will precisely focus on residues specifically affecting pathogenic species, or pathogen-specific residues. Such design of inhibitors would give a more customized and focused treatment than antibiotics, which are currently the only choice.

Numerous alanine mutations exhibit markedly increased adherence propensities (Fig. 8). These alanine mutations may significantly increase hydrophobicity, thus indicating that the interaction between Gp340 and *S. mutans* surface proteins is facilitated by hydrophobic forces. It is tempting to speculate that the complementary surfaces on Gp340’s SRCR domains could explain the mechanism behind enhanced adhesion, although this remains speculative at present. Further studies with mutations on the SRCR domains of Gp340 would provide conclusive evidence.

The AgI/II-family of proteins mediate streptococcal adherence to collagen, fibronectin, laminin, and other ECM proteins; however, not all members bind to the same ligands, suggesting species-specific targeting of ECMs (41). This becomes more fascinating outside of the oral cavity, where oral streptococcal interactions with ECM proteins and other microbes occur, specifically in the colonization of vital organs (1, 9). Oral streptococci are thought to gain access through periodontal damage and/or are facilitated by other gum damages/tears that take place through dental procedures (42). For example, *S. gordonii* is an oral commensal but is also a major colonizer of the heart valves in infective endocarditis (43). The capacity of SspB to bind to fibronectin aids in its invasion of the bloodstream and infection of heart valves (44). Small molecule compounds that inhibit such interactions will keep these organisms out of difficult-to-treat sites of illness. The affinities of AgI/II-family proteins vary by species, which affects the pathogenicity of oral streptococci through attachment to host surfaces, interactions with other bacteria, and the creation of multispecies biofilms at infection sites. Given these adhesins‘ propensity to attach to a variety of ligands, it would be worthwhile to investigate and determine whether overlapping binding sites exist. This would be an essential investigation, as it might lead to the development of multifunctional inhibitors that are species-specific and focused on specific disease conditions. Our examination of both commensal and pathogenic strains in the oral cavity has identified specific residues within these highly homologous proteins, demonstrating the potential for the targeted discovery of species-specific therapies.

## MATERIALS AND METHODS

### Cloning of V^SspB^

The SspB gene, a gift from Prof. Richard Lamont’s laboratory, was used as the template for cloning V^SspB^ and V^SspB^-noh, while V^AgI/II^ was subcloned from our previously reported A3VP1 construct of AgI/II. Primers (forward) with a *NcoI* site and (reverse) with the *NotI* site and a TEV cleavage peptide sequence “ENYFQGS” to remove the histidine tag at the C-terminus were designed to clone the V^SspB^ and V^SspB^-noh inserts into the pET23d vector (Novagen, Inc), whereas V^AgI/II^ was cloned using *NcoI* and *XhoI* restriction enzymes. All constructs have a C-terminal hexa-histidine tag incorporated for affinity purification. PCR amplifications were carried out using the PhusionTM DNA polymerase, the products were purified, and both the vector and the fragments were subjected to digestion by *NcoI* and *NotI/XhoI*. They were then ligated using T4 DNA ligase (NEB) and thereafter, transformed into *Escherichia coli* DH5α cells and plated on Luria Bertani (LB) ampicillin plates for overnight incubation at 37°C. Colonies were picked and grown in 5 mL LB broth with ampicillin overnight at 37°C, and then, the plasmids were extracted. These plasmids were subsequently sequenced to confirm the presence of the V^SspB^, V^SspB^-noh, and V^AgI/II^ inserts at the University of Alabama at Birmingham (UAB) Heflin Center for Genomic Sciences. After confirmation, the plasmids were transformed into *E. coli* BL21(DE3) cells for protein expression. Primers (forward and reverse) used for cloning of V^SspB^, V^SspB^-noh, and V^AgI/II^ are listed in Table 1.

### Protein expression and purification

*E. coli* BL21(DE3) cells harboring the V^SspB^ plasmid were inoculated into a 20 mL Terrific Broth (TB) starter culture overnight at 37°C. The following day, the cells grown in the starter culture were transferred to shaker flasks containing 1 L of TB and grown until their OD_600_ reached 1.0, at which point they were induced with 1 mM isopropyl-beta-D-thiogalactopyranoside and grown for 5 h at 30°C. At this point, the 1 L of TB broth was supplemented with an additional dose of ampicillin and grown overnight at 18°C, after which the cells were harvested by centrifugation at 5,000 × *g* for 20 mins using a Beckman Avanti JL-25 centrifuge. The cell pellets were suspended in nickel affinity column binding buffer (50 mM Tris, pH 8.0, and 500 mM sodium chloride) augmented with a cOmplete EDTA-free protease inhibitor (Roche). Cells were ruptured by sonication (Fisher brand Sonicator) for a total of 5 min while maintaining a maximum temperature of 10°C. They were thereafter subjected to ultracentrifugation at 35,000 RPM for 1 h using a Ti70 rotor, where the supernatant was collected and filtered through a 0.22 micron filter before being loaded onto a 20 mL HisPrep Nickel Column (GE Healthcare, Inc.). Using a step gradient of 50 mM imidazole, non-specifically bound proteins were gently removed from the column, and then the bound protein was eluted with a 50–300 mM imidazole gradient. The protein fractions were selected based on purity on an SDS-PAGE gel, pooled together, and dialyzed overnight into Mono Q buffer (50 mM Tris, pH 8.0, 50 mM sodium chloride, and 1 mM EDTA). This dialyzed sample was loaded onto a Mono Q column (GE Healthcare, Inc.), and the protein was eluted with a 0–400 mM NaCl gradient. The purest single-banded fractions as identified by SDS-PAGE gels were then pooled and concentrated under 55 psi nitrogen gas using an Amicon stirring concentrator. Protein concentration was measured using a modified method described elsewhere (45). Similar methods were used with minor modifications in the purification of V^SspB^-noh and V^AgI/II^.

### Crystallization and data collection

V^SspB^ (25 mg/mL) was initially subjected to the Hampton Research Index Crystallization HT screen, and crystals were obtained in condition #24 (2.8 M sodium acetate tri-hydrate, pH 7.0). This condition was further optimized, and large crystals suitable for data collection were obtained in 3.0 M sodium acetate trihydrate and 100 mM Hepes, pH 7.0. Similarly, crystals for V^SspB^-noh were obtained using 20%–25% PEG 8000 as precipitant, in 0.1 M succinic acid pH 5.0–5.5 and 100 mM MgCl_2_. Briefly, 1 µL of reservoir solution was mixed with 1 µL of concentrated protein (V^SspB^-noh at 20.0 mg/mL and V^SspB^ at 25 mg/mL) in a hanging drop vapor diffusion setup using Linbro boxes. Finally, crystals of V^AgI/II^ (11.5 mg/mL) were obtained using 25% PEG 4000, 650 mM Li_2_SO_4_, and 50 mM Tris pH 7.5. Diffraction-quality V^SspB^, V^SspB^-noh, and V^AgI/II^ crystals were flash frozen in buffer of the crystallization condition supplemented by 15% ethylene glycol, 20% glycerol, and 12% ethylene glycol, respectively. Diffraction data for V^SspB^ and V^SspB^-noh were collected on our Rigaku MicroMax 007HF home source equipped with a Dectris Pilatus 200K detector, while V^AgI/II^ was collected at the NE-CAT 24-ID synchrotron beamline located at the Advanced Photon Source of the Argonne National Labs. Data were integrated, merged, and scaled with HKL3000 (46). Data collection parameters are listed in Table 6.

**TABLE 6:** Crystallographic parameters

Parameter	V^SspB^ (6Q2K)	V^SspB^-noh (6Q2L)	V^AgI/II^ (6TZL)
Data collection			
Space group	P4_3_2_1_2	P2_1_	P2_1_2_1_2_1_
Unit cell parameters	a = b = 121.51 Å c = 50.75 Å	a = 60.20 Å b = 47.68 Å c = 64.67 Å β = 113.6°	a = 65.97 Å b = 133.33 Å c = 246.07 Å
Resolution (Å)	30.00–2.00 (2.03–2.00)	50.00–1.80 (1.83–1.80)	50.00–1.60 (1.63–1.60)
Matthews coefficient *V*_*M*_	2.50	2.45	3.02
Unique reflections	25,664 (1,071)	31,075 (1,321)	285,302 (14,076)
Completeness (%)	97.7 (82.8)	98.9 (83.9)	99.9 (100)
Multiplicity	15.5 (3.1)	3.3 (1.5)	4.1 (4.1)
*R*_merge_ (%)	11.8 (44.5)	8.4 (41.9)	5.6 (70.2)
*R*_pim_ (%)	2.7 (27.5)	4.7 (38.0)	3.1 (39.6)
CC_1/2_	0.839 (2.03–2.00 Å)	0.714 (1.83–1.80 Å)	0.690 (1.63–1.60 Å)
CC^*^	0.955 (2.03–2.00 Å)	0.913 (1.83–1.80 Å)	0.904 (1.63–1.60 Å)
I/sI	20.1 (2.4)	19.1 (1.6)	24.3 (1.5)
Refinement			
Resolution (Å)	29.47–2.00 (2.05–2.00)	37.15–1.80 (1.86–1.80)	34.96–1.60 (1.64–1.60)
No. of reflections	25,579 (1,608)	31,061 (2,821)	285,097 (20,707)
Completeness	97.4 (84.8)	98.8 (90.6)	99.8 (99.0)
*R*_work_ (%)	19.4 (26.9)	19.5 (30.3)	18.0 (26.8)
*R*_free_ (%)	23.6 (32.6)	23.2 (30.2)	19.3 (27.4)
B-factors			
Wilson B (Å^2^)	27.7	17.4	19.9
Average B (Å^2^)	31.4 (2,752 atoms)	22.5 (2,721 atoms)	26.7 (14,201 atoms)
Protein (Å^2^)	30.4 (2,470 atoms)	21.1 (2,363 atoms)	26.9 (12,257 atoms)
Ion (Å^2^)	49.2 (Ca^2+^)	13.5 (Mg^2+^)	–^*a*^
GOL (Å^2^)	–	20.2	–
SO_4_ (Å^2^)	–	–	70.8 (30 atoms)
Water (Å^2^)	39.8 (281 atoms)	32.1 (351 atoms)	36.1 (1,914 atoms)
Model statistics			
Rmsd bonds (Å)	0.006	0.009	0.012
Rmsd angles (Å)	1.10	1.33	1.64
CC (Fo-Fc)	0.95	0.95	0.97
CC (Fo-Fc free)	0.94	0.93	0.96
Ramachandran	97% favored 3% allowed (1 outlier)	97% favored 3% allowed (1 outlier)	98.1% favored 1.9% allowed (0 outliers)
Clash score	0.00	0.10	1.90
Molprobity score	0.66	0.86	0.95

^*a*^ –, no data.

### Crystal structure determination and refinement

The structures for V^SspB^ and V^SspB^-noh were solved by molecular replacement using HKL3000 with the previously reported structure of the SspB V-domain (32) (PDB2WD6) as initial search model. Refinement was carried out by a combination of Refmac5 (47) in CCP4 (48) and Phenix (49), and for V^SspB^-noh, we performed isotropic B-factor refinement with TLS (50). For model building, we used Coot (51). Figures were created with PyMol (52). To assess the model quality, we used a combination of validations in Phenix and the Worldwide PDB (https://validate-rcsb-2.wwpdb.org/). The validation consists of Molprobity (53) results (Geometry, Clashes, etc.) for the protein residues and fitness to the electron density for protein residues, water molecules, and metal ions. The conserved Ca^2+^ site in V^SspB^ and the presence of Mg^2+^ instead of Ca^2+^ in V^SspB^-noh were verified using the CheckMyMetal web server (https://csgid.org/csgid/metal_sites/). The coordinates have been deposited into the PDB (RCSB) where V^SspB^ and V^SspB^-noh are represented by PDB6Q2K and PDB6Q2L, respectively. Similar methods were used to resolve the structure of V^AgI/II^, where the initial model was derived from the A_3_VP_1_ structure (PDB3IPK) and deposited as PDB6TZL. Refinement statistics are shown in Table 6.

### Design and synthesis of peptide PepCD1^SRCR^

The TEV peptide (ENLYFQGAAALE; TEV^Pep^) bound within the V^SspB^ pocket showed both structural and sequence homology to residues DTNDANVVCRQL in the SRCR domains (Fig. S1). We synthesized the following sequence, “ETNDANVVARQL,” where the first amino acid “D” was substituted with an “E” to enable the N-terminal fluorescein amidite attachment, and the C was replaced with “A to avoid disulfide formations.

### SPR studies

The binding affinity coefficients of PepCD1^SRCR^ with recombinantly expressed V^SspB^, V^AgI/II^, and V^GbpC^ were determined using SPR on a BIAcore 2000 (GE Healthcare, Inc). Briefly, the chip surface was prepared by immobilizing V^SspB^-noh, V^AgI/II^, and V^GbpC^ on a CM5 chip using amine coupling. The analyte PepCD1^SRCR^ was injected at various concentrations (10–80 μM) over this prepared surface for 120 s to measure association, followed by dissociation for 600 s, with the resultant sensorgrams being recorded (Fig. 6). Each experiment was carried out in triplicate, and the sensorgrams were analyzed using the BIAevaluation software, where both residuals and Chi^2^ values were refined toward convergence. The results are presented in Table 5. Our binding experiments with V^SspB^ did not display binding, and we surmised that the bound TEV^Pep^ would have prevented the binding of PepCD1^SRCR^.

To determine binding of the alanine-substituted mutations of V^SspB^, V^AgI/II^, and V^GbpC^, SPR studies were performed using immobilized SRCR_1_ that was recombinantly expressed and purified from insect cells as previously described (54). Briefly, SRCR_1_ ligand was immobilized on a CM5 chip using ethanolamine chemistry, and the chip surface was prepared for binding experiments. V-region analytes were injected over SRCR_1_ at various concentrations (serial dilutions within 2–8 µM), dissociation was measured for 600 s following injects, and the sensorgrams were recorded using a BIAcore T200. The sensorgrams were fitted using Biacore Insight Evaluation software. The binding percentage was determined with the WT established at 100%, and each mutant’s binding was expressed as a percentage of the WT at a ligand concentration of 8 µM. All studies were conducted in triplicate.

### Biofilm assays

WT *S. mutans* UA159 (*SM*), AgI/II-deletion mutant (*SM*-ΔAgI/II) (30), and GbpC-deletion mutant (*SM*-ΔGbpC) (30) with various concentrations of PepCD1^SRCR^ were grown in TSYE broth with 1% sucrose for 16 h on an unconditioned surface, after which biofilm biomass was quantified by staining with crystal violet dye. The optical density was measured at 562 nm, as described previously (55, 56). Results were plotted as bar graphs (Fig. 7). The peptide DSRIRMGFDFSK was used as a negative control (100 μg/mL). *S. gordonii DL1* WT biofilm assay was performed similar to *S. mutans* with modifications of 50% TSYE broth and 1% glucose.

### Statistical analysis

All statistical analyses reported in this article were assessed using GraphPad Prism with one-way analysis of variance (ANOVA) and the Tukey-Kramer test, where significance labels are denoted as *P < 0.05, **P < 0.01, ***P < 0.001, ****P < 0.0001.

## SUPPLEMENTAL MATERIAL

Fig. S1 iai.00467-25-s0001.tiff Model of SRCR highlighting PepCD1^SRCR^ peptide.

Fig. S2 iai.00467-25-s0002.tiff SPR sensorgrams of V^AgI/II^ alanine substituted mutant binding to immobilized SRCR_1_ using BIAcore T200.

Fig. S3 iai.00467-25-s0003.tiff SPR sensorgrams of V^GbpC^ alanine substituted mutant binding to immobilized SRCR_1_ using BIAcore T200.

Fig. S4 iai.00467-25-s0004.tiff SPR sensorgrams of V^SspB^ alanine substituted mutant binding to immobilized SRCR_1_ using BIAcore T200.

Fig. S5 iai.00467-25-s0005.tiff Studies on biofilm formation using wild type *S. gordonii* and *S. gordonii* ΔSspB indicate that SspB does not significantly influence biofilm development*.*

Supplemental material iai.00467-25-s0006.docx Supplemental figure legends.

ASM does not own the copyrights to Supplemental Material that may be linked to, or accessed through, an article. The authors have granted ASM a non-exclusive, world-wide license to publish the Supplemental Material files. Please contact the corresponding author directly for reuse.
