# Supplementary figures and images for: The structure of Streptococcus gordonii surface protein SspB in complex with TEV peptide provides clues to oral streptococcal adherence to salivary agglutinin
Source: Infect Immun. 2026 Feb 4;94(3):e00467-25. doi: 10.1128/iai.00467-25 (PMC12974128; doi:10.1128/iai.00467-25)

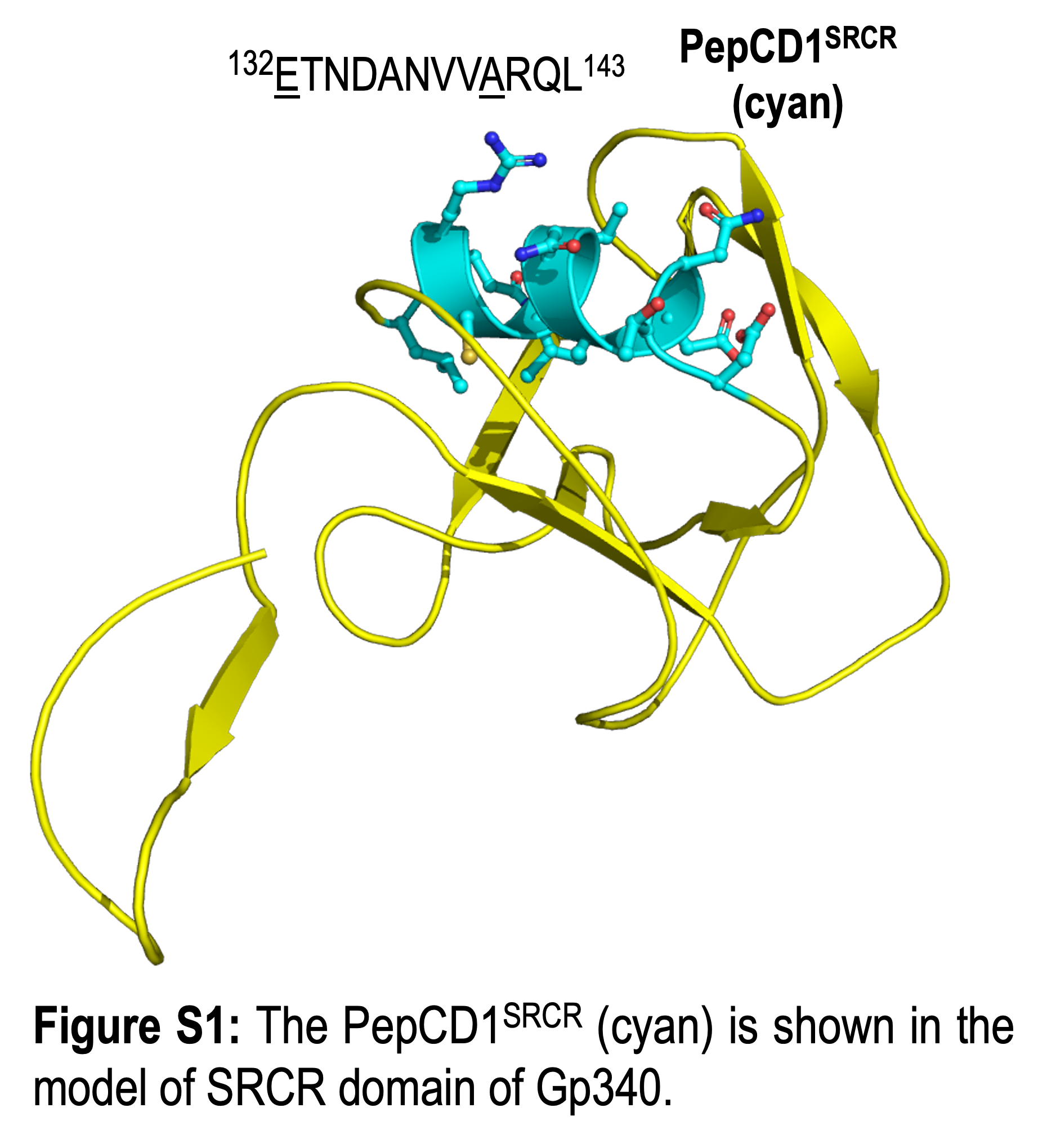

Supplement: Fig. S1 — Model of SRCR highlighting PepCD1SRCR peptide. [file iai.00467-25-s0001.tiff]

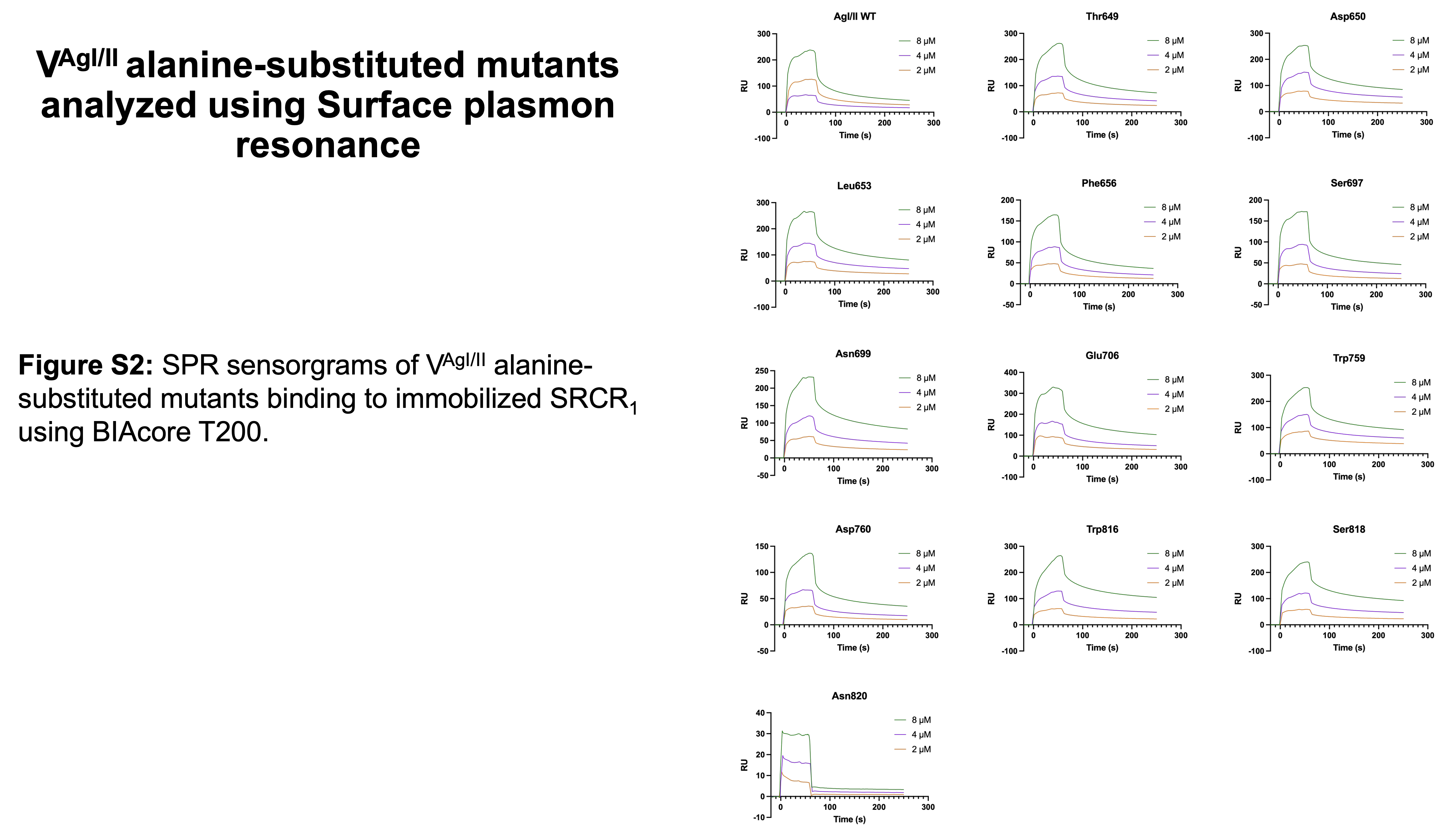

Supplement: Fig. S2 — SPR sensorgrams of VAgI/II alanine substituted mutant binding to immobilized SRCR1 using BIAcore T200. [file iai.00467-25-s0002.tiff]

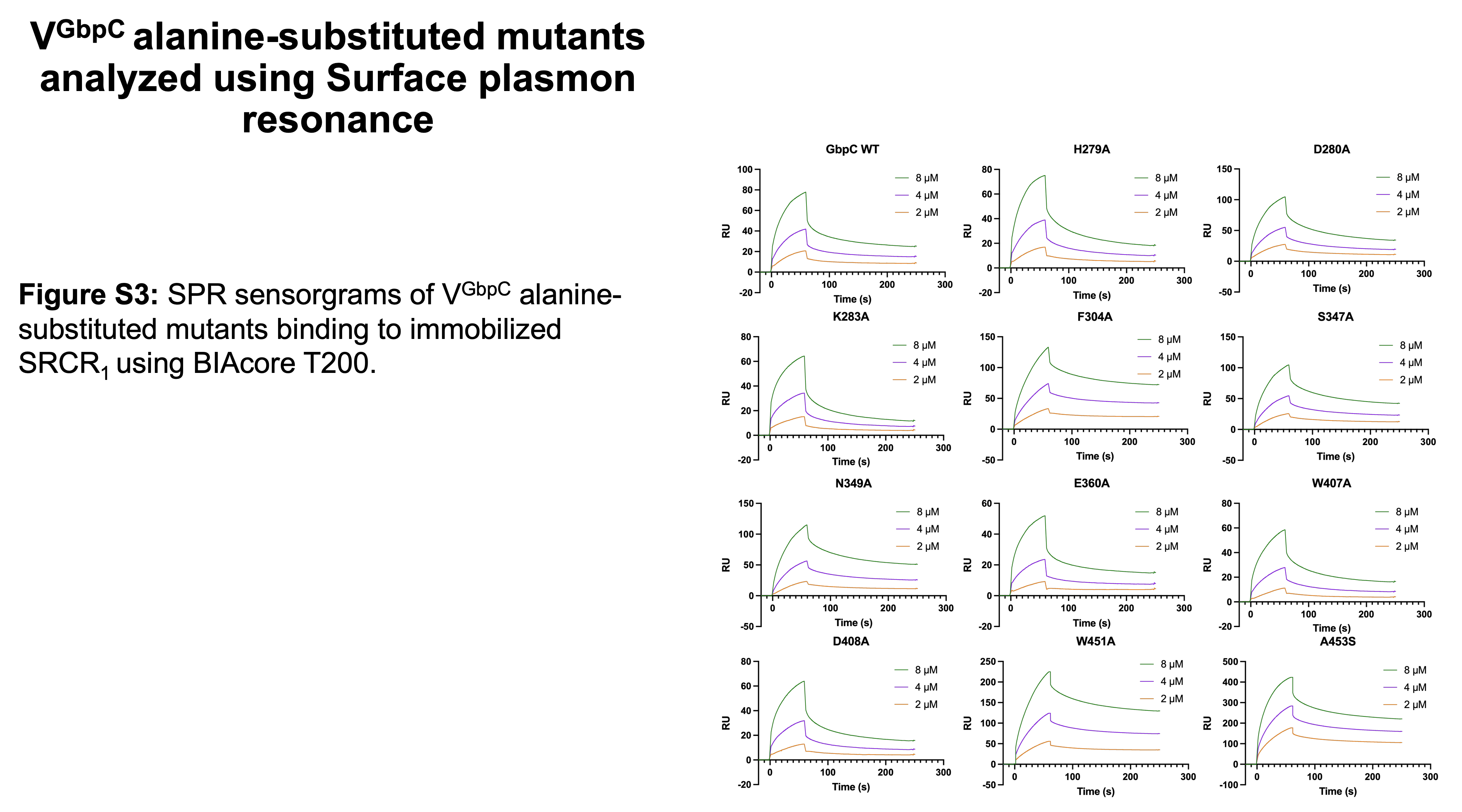

Supplement: Fig. S3 — SPR sensorgrams of VGbpC alanine substituted mutant binding to immobilized SRCR1 using BIAcore T200. [file iai.00467-25-s0003.tiff]

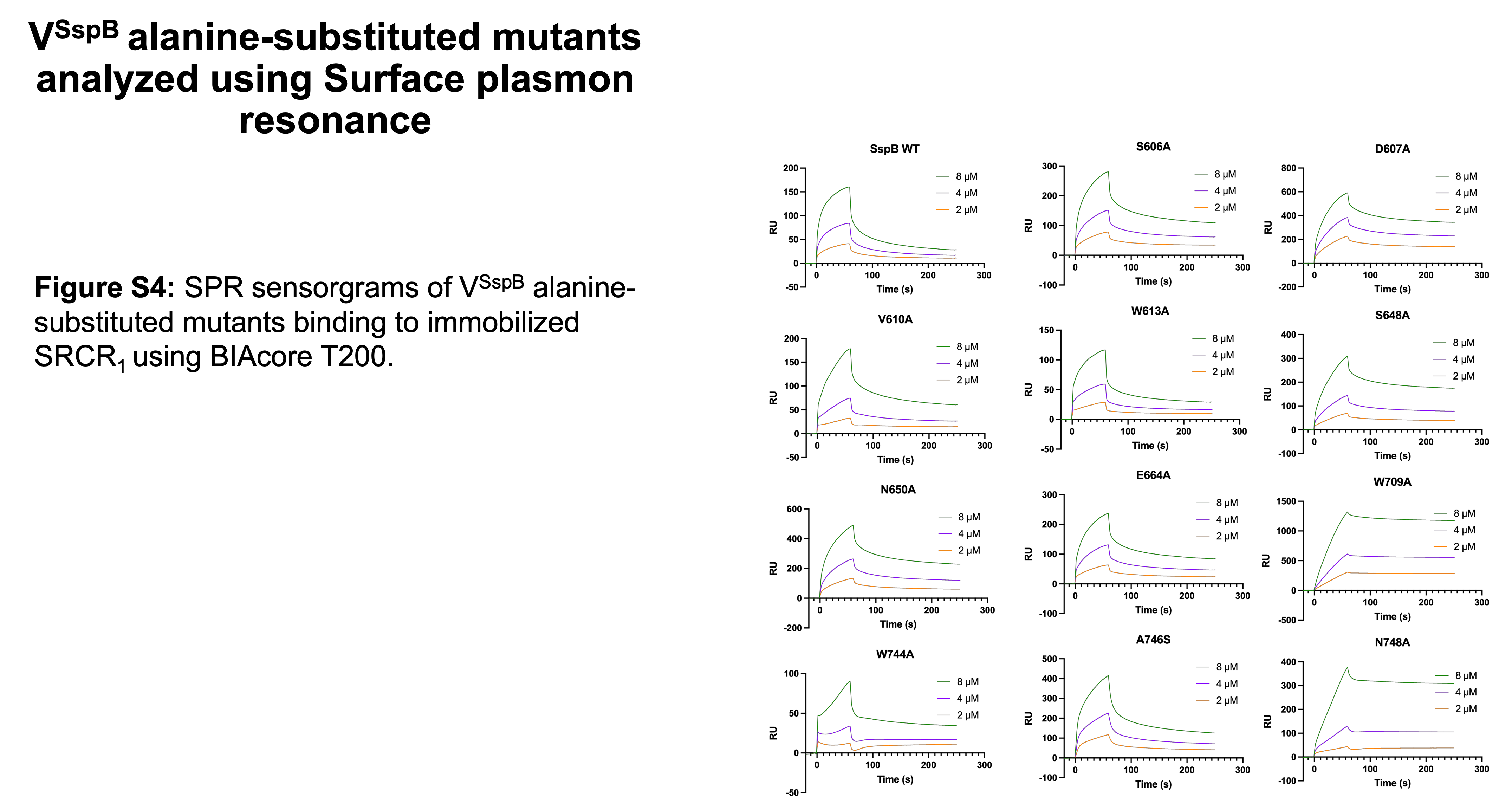

Supplement: Fig. S4 — SPR sensorgrams of VSspB alanine substituted mutant binding to immobilized SRCR1 using BIAcore T200. [file iai.00467-25-s0004.tiff]

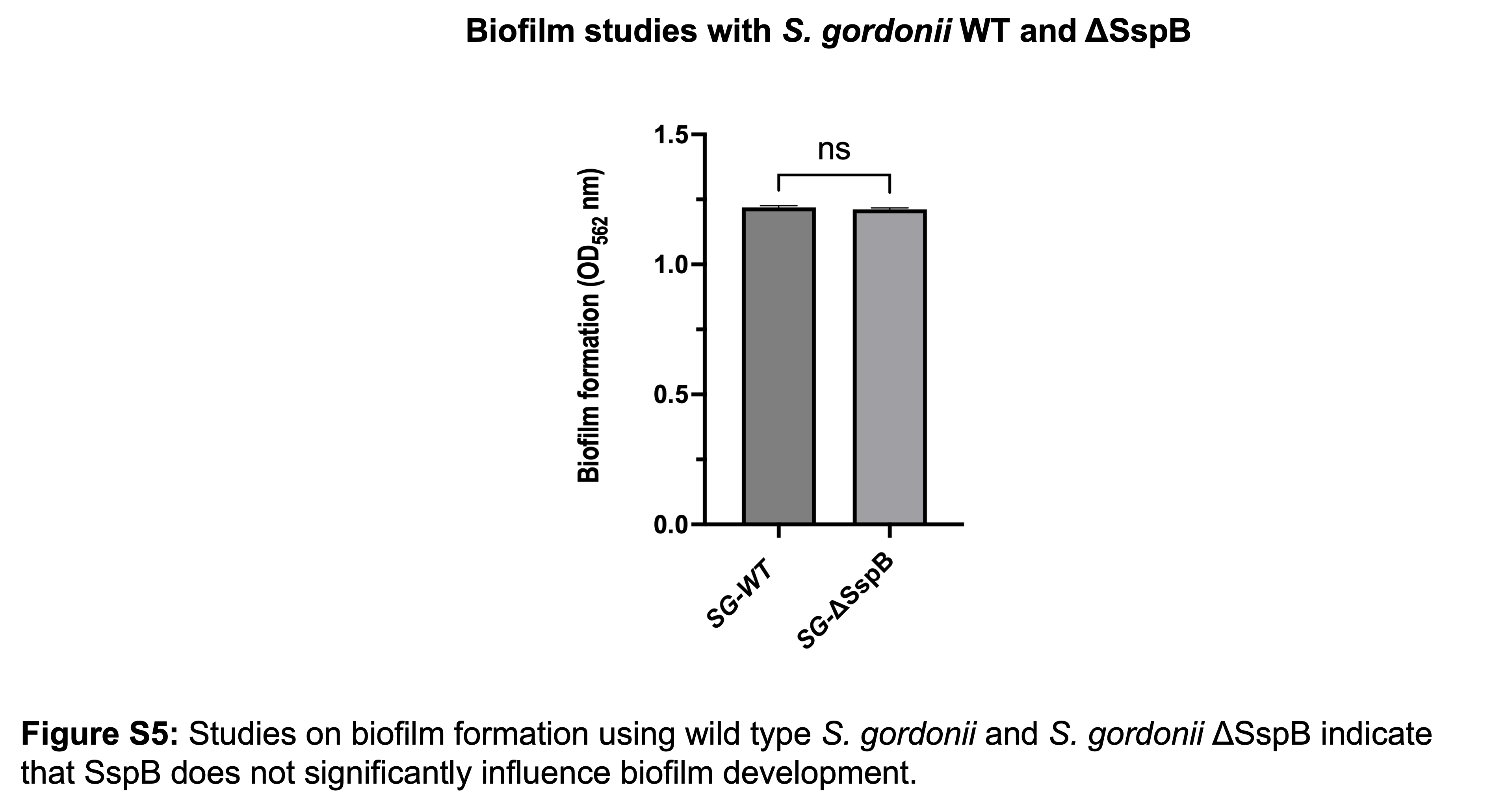

Supplement: Fig. S5 — Studies on biofilm formation using wild type S. gordonii and S. gordonii ΔSspB indicate that SspB does not significantly influence biofilm development. [file iai.00467-25-s0005.tiff]
