# Supplementary material for: The structure of Streptococcus gordonii surface protein SspB in complex with TEV peptide provides clues to oral streptococcal adherence to salivary agglutinin
Source: Infect Immun. 2026 Feb 4;94(3):e00467-25. doi: 10.1128/iai.00467-25 (PMC12974128; doi:10.1128/iai.00467-25)
Supplement: Supplemental material — Supplemental figure legends. [file iai.00467-25-s0006.docx]

**Supplemental Figure Legends**

**Figure S1**: The PepCD1^SRCR^ (cyan) is shown in the model of SRCR domain of Gp340.

**Figure S2**: SPR sensorgrams of V^AgI/II^ alanine-substituted mutants binding to immobilized SRCR_1_ using BIAcore T200.

**Figure S3**: SPR sensorgrams of V^GbpC^ alanine-substituted mutants binding to immobilized SRCR_1_ using BIAcore T200.

**Figure S4**: SPR sensorgrams of V^SspB^ alanine-substituted mutants binding to immobilized SRCR_1_ using BIAcore T200.

**Figure S5:** Studies on biofilm formation using wild type *S. gordonii* and *S. gordonii* ΔSspB indicate that SspB does not significantly influence biofilm development.
